# Knowledge, attitudes and factors associated with uptake of modern contraceptive methods among young women living with disabilities in Botswana

**DOI:** 10.4102/phcfm.v18i1.4977

**Published:** 2026-02-02

**Authors:** Charity S. Moses, Olubukola Adesina, Lucia M. Mupara

**Affiliations:** 1 Department of Reproductive Health Sciences, Faculty of Obstetrics and Gynaecology, Pan African University Life and Earth Sciences Institute (Including Health and Agriculture), Ibadan, Nigeria; 2 Department of Obstetrics and Gynaecology, Faculty of Reproductive Health Sciences, Pan African University Life and Earth Sciences Institute (Including Health and Agriculture), Ibadan, Nigeria; 3 Department of Obstetrics and Gynaecology, College of Medicine, University College Hospital, Ibadan, Nigeria; 4 National Institute of Maternal and Child Health, University of Ibadan, Ibadan, Nigeria; 5 Department of Community Health, Partners in Health, Monrovia, Liberia

**Keywords:** disabilities, Botswana, modern contraceptives, reproductive health, adolescents, young women, health care, sexually transmitted infections

## Abstract

**Background:**

Young women living with disabilities in Botswana face significant challenges in accessing reproductive health services, including modern contraceptive methods, yet their unique needs and barriers to uptake remain underexplored.

**Aim:**

The aim of this study is to assess the knowledge, attitudes and factors associated with the uptake of modern contraceptive methods among young women living with disabilities in Botswana.

**Setting:**

This study was conducted in eight districts in Botswana, within organisations that offer services to people living with disabilities.

**Methods:**

This cross-sectional study was conducted among young women (10–30 years) living with disabilities, recruited through non-probability purposive sampling from disability service organisations. Using an interviewer administered tool, data collected was analysed using STATA 15.

**Results:**

Among the 349 participants, the pattern of disabilities were deaf or hard of hearing (36.68%), physical disability (30.09%) and albinism (1.43%). The majority (71.6%) were aware of contraceptive methods, and 69.6% received information from health personnel. Of those, 60.4% knew oral pills, and 81.6% linked modern contraceptives to preventing sexually transmitted infections. About 38.3% reported using modern contraceptives, mainly male condoms. Stigmatisation fears emerged as a significant barrier (36.0%).

**Conclusion:**

Knowledge, gaps and unfavourable attitudes towards contraception were evident suggesting a need for interventions to meet needs of young people living with disabilities.

**Contribution:**

The study provides critical insights into the knowledge, attitudes and barriers affecting the use of modern contraceptives among young women living with disabilies in Botswana, offering evidence to inform more inclusive healthcare policies and programmes.

## Introduction

Disability, according to the World Health Organization, is the outcome of interactions between individuals with a health condition and different environmental and personal factors such as unfavourable attitudes, inaccessible public spaces and modes of transportation and limited social support.^1^ It is estimated that over 1 billion individuals worldwide suffer from a disability. This corresponds to over 15% of the global population, with up to 190 million (3.8%) adults over the age of 15 years experiencing severe functional impairments that frequently necessitate health care services.^2^ Research shows that people living with disabilities face a number of significant obstacles, including poverty, social and economic marginalisation and limited access to public spaces, transportation and information.^3^ Disability has human rights implications. Individuals living with disabilities encounter multiple types of discrimination because of their disability, which often overlaps with discrimination based on gender and age, among other characteristics. These violations include acts of violence, abuse, prejudice and disrespect.^4^

People living with disabilities, like everyone else, have demands of sexual reproductive health (SRH). Yet, they frequently encounter obstacles in accessing services and information.^5^ In spite of advancements, adolescents and women living with disabilities remain vulnerable because they are often denied access to SRH services.^6^ Access to competent, comprehensive and discrimination-free SRH services, information and education is severely impeded for young women and adolescents living with disabilities.^6^ As a result, young people living with disabilities are exposed to sex-related violence, harassment, human immunodeficiency virus and acquired immunodeficiency syndrome (HIV and AIDS), unintended pregnancies and other sexually transmitted infections (STIs) that constitute a serious threat to their quality of life, health and personal growth.^7^

In Botswana, it has been estimated that 4.5% of the population is disabled.^8^ And although Botswana has one of the highest rate of contraceptive prevalence in Africa (67.4%),^8^ no study has examined the prevalence, knowledge, attitude and uptake of modern contraceptives among young people living with disabilities in Botswana.^8^ As a result, it is unknown how young women in Botswana who live with disabilities use, understand or feel about contraception. Prior research on contraceptive methods in Botswana was done on the general public, and it was found that 44% of women said they were pregnant against their will.^9^ The aim of this study was to assess the prevalence and knowledge, attitudes and factors associated with the uptake of modern contraceptive methods among young women living with disabilities in Botswana.

## Research methods and design

### Study design

This was a cross-sectional analytical study conducted among girls and young women living with disabilities in Botswana.

### Setting

The study was conducted in organisations in Botswana that offer services to people living with disabilities. These organisations were registered with the Botswana Council of Disabilities and served as the primary location for recruitment.

### Study population and sampling strategy

The study population comprised young adults, girls and women aged between the ages of 10 years and 30 years living with disabilities accessing services at centres providing care to this population. The inclusion criteria were girls and women aged between the age of 10 years and 30 years with vision impairment, deaf or hard of hearing, albinism, physical disability and those who were able to consent or provide assent. Participants aged 10–15 years were included to capture early awareness, knowledge and attitudes formed during the initial stages of sexual health education, even if they had limited direct experience accessing reproductive health services. The exclusion criteria included those with mental health conditions, intellectual disability, autism spectrum disorder, women above 18 years who did not provide consent and girls less than 18 years who did not assent to the study or whose care givers did not consent.

The sample size was calculated using a prevalence 33.7% from a study in Ethiopia^10^ (*n* = 10) with a 95% confidence interval (CI) and a degree of precision of 5 (standard deviation [s.d.]). A total of 349 women living with disabilities were interviewed in the study. The study used non-probability purposive sampling method. First of all, all the organisations registered with Botswana Council of Disabilities were listed and approached for permission. Women and girls in the organisations that gave permission who met the inclusion criteria were approached. They were recruited after they had signed the informed consent form or they assented to the study as maybe appropriate.

### Data collection

A structured interviewer administered questionnaire was used for data collection. The questionnaire was developed following careful review. After being translated into the easily understood Setswana language, the questions were back-translated into English. Information collected included the socio-demographic variables, factors affecting uptake of contraceptive methods, the knowledge and attitude on contraceptive methods and the barriers and facilitators of uptake of contraceptive methods among young women living with disabilities. The study’s dependent variable was the uptake of modern contraceptive methods, and the independent variables were knowledge and attitudes towards the uptake of modern contraceptive methods among young women living with disabilities and the socio-demographic variables of the study participants.

### Data analysis

The quantitative data collected were entered into Microsoft Office Excel 2019. The data were analysed using STATA 15 (STATA Corporation, 4905, Lakeway River, College Station, Texas, United States). Frequency tables with percentages were constructed. For the analysis, both descriptive and inferential statistics were used. To determine the significance of the association between the variables, the Pearson Chi-square test was employed. Significant values were those with a 95% CI and a *p*-value less than 0.05.

### Ethical considerations

Ethical approval was obtained from the Joint University of Ibadan/University College Hospital Ethics Committee (COMREC) with study approval number UI/EC/23/0101. Further approval was obtained from the Botswana Ministry of Health (Reference No: HPRD: 6/14/1), the Ministry for State President (REF: OP 5/59/8 XXXIII(24) and the Ministry of Education and Skills Development (REF: DPRS 7/1/5 XXXVIII(40).

Written permission was sought from the organisations. An informed written consent was obtained from each participant or their care providers or assent from appropriate participants.

Data confidentiality was strictly maintained. Each participant was assigned a unique identifier code on the questionnaire to ensure anonymity. All collected information was coded, and no personally identifiable details, such as names, were recorded. The data were solely used for the research purposes, and any reports or publications resulting from this study will not contain any identifying information.

To promote honest and candid responses, interviews were conducted in private settings by trained interviewers who assured participants of confidentiality and the voluntary nature of their participation.

The study involved human participants, and all procedures were carried out in accordance with ethical standards.

## Results

### Socio-demographic characteristics of study participants

**FIGURE 1:**
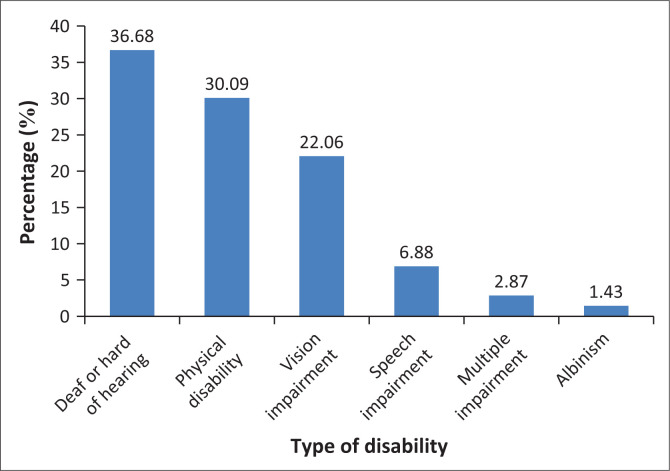
Types of disabilities in percentages.

A total of 349 women living with disabilities were found to be eligible, all of whom consented to participate and complete the interviewed. This resulted in 100% consent and response rate. The participants’ mean (± s.d.) age was 20.5 ± 6.1 years. About one-third of the participants were aged 16–20 years and another third aged 25–30 years, the majority (91.1%) were single and 65.9% were students with 35.2%, having junior high school level of education. As shown in Figure 1, the most common disabilities identified were hearing impairments (36.7%) and physical impairments (30.1%) (Table 1).

**TABLE 1:** Participants’ socio-demographic characteristics.

Variable	Frequency		Knowledge (*n* = 250)		No knowledge (*n* = 99)		*p*-value	*χ* ^2^
	*n*	%	*n*	%	*n*	%		
**Age (years)**							< 0.0001	183.1
10–15	64	18.3	3	1.20	61	61.6	-	-
16–20	115	32.9	102	40.80	13	13.1	-	-
21–25	56	16.1	56	22.40	0	0.0	-	-
26–30	114	32.7	89	35.60	25	25.3	-	-
**Marital status**							0.0010	13.5
Married	23	6.6	23	9.20	0	0.0	-	-
Single	318	91.1	219	87.60	99	100.0	-	-
Widowed	8	2.3	8	3.20	0	0.0	-	-
**Educational level**							< 0.0001	94.3
Lower primary (1–4)	74	21.2	30	12.00	44	44.4	-	-
Upper primary (5–7)	85	24.4	46	18.40	39	39.4	-	-
Junior secondary	123	35.2	115	46.00	8	8.1	-	-
Senior secondary	35	10.0	35	14.00	0	0.0	-	-
Tertiary	8	2.3	8	3.20	0	0.0	-	-
Vocational training	24	6.9	16	6.40	8	8.1	-	-
**Occupation**							0.0080	15.6
Unemployed	84	24.1	59	23.60	25	25.3	-	-
Student	230	65.9	156	62.40	74	74.6	-	-
House wife or homemaker	8	2.3	8	3.20	0	0.0	-	-
Small business person	22	6.3	22	8.80	0	0.0	-	-
Junior civil servant	5	1.4	5	2.00	0	0.0	-	-
**Place of residence**							< 0.0001	39.9
Rural	104	29.8	51	20.40	53	53.5	-	-
Urban	186	53.3	156	62.40	30	30.3	-	-
Settlement	59	16.9	43	17.20	16	16.7	-	-
**Region of residence**							< 0.0001	193.2
South East or Southern	100	28.6	92	36.80	8	8.1	-	-
Kweneng	34	9.7	34	13.60	0	0.0	-	-
Kgatleng	58	16.6	58	23.20	0	0.0	-	-
Central	24	6.9	0	0.00	24	24.2	-	-
Ngwaketsi or Kgalagadi	71	20.3	52	20.80	19	19.2	-	-
North East	34	9.7	5	2.00	29	29.3	-	-
North West or Ngamiland	28	8.0	9	3.60	19	19.2	-	-
**Religion**							0.0360***^ns^***	8.6
African traditional religion	7	2.0	7	2.80	0	0.0	-	-
Christianity	324	92.8	233	93.20	91	91.9	-	-
Non-religious	15	4.3	7	2.80	8	8.1	-	-
**Type of disability**							< 0.0001	107.4
Albinism	5	1.4	1	0.40	4	4.0	-	-
Deaf or hard of hearing	128	36.7	56	22.40	72	72.7	-	-
Physical disability	105	30.1	89	35.60	16	16.2	-	-
Speech impairment	24	6.9	23	9.20	1	1.0	-	-
Vision impairment	77	22.1	76	30.40	1	1.0	-	-
Multiple impairments	10	2.9	5	2.00	5	5.1	-	-

Note: Difference at 5% significance level (i.e. *p* < 0.05) (ns).

*ns*, no significant.

### Knowledge on modern contraceptive methods

The majority of the participants, 250 (71.6%), have heard of contraceptive methods. Among those who have heard, 69.6% cited health personnel as their main source of information followed by 45.2 of those who have heard from the school with the least being print media at 1.2%. Among the participants who were knowledgeable about contraception, 60.4% acknowledged oral pills as one of the methods of contraception, followed by injectables at 59.2 and male condoms at 56.0%. Table 2 shows the participants’ knowledge about modern contraception.

**TABLE 2:** Knowledge of participants about modern contraceptive methods.

Variable	*n*	%
**Heard of contraceptive method**		
Yes	250	71.4
No	99	28.4
**Source of information**		
Health personnel	174	69.6
Family	30	12.0
Friends	50	20.0
School	113	45.2
Radio	48	19.2
Television	19	7.6
Print media	3	1.2
**Modern contraceptive known**		
IUD	15	6.0
Implants	98	39.2
Male condoms	140	56.0
Female condoms	14	5.6
Injectables	148	59.2
Pills	151	60.4
**Reasons for using contraceptives**		
Limiting number of children	104	41.6
Preventing pregnancy	79	31.6
Stopping child birth	130	52.0
The spacing of birth interval	69	27.6
Prevent STI	204	81.6

STI, sexually transmitted infection; IUD, intrauterine device.

### Attitudes towards contraception use

When the attitude towards contraception was assessed, 40% – 60% of the participants believe that contraception makes women unhealthy, interferes with sexual relationship and results in infertility and that women are more responsible in the use of modern contraceptives. Most believe that contraceptives can prevent STIs (69.2%), prevent pregnancies (78.5%) and help in child spacing (46.4%) (Table 3).

**TABLE 3:** Attitudes of participants towards the use of modern contraception methods.

Opinion towards contraception use	Strongly agree		Agree		Do not know		Disagree		Strongly disagree	
	*n*	%	*n*	%	*n*	%	*n*	%	*n*	%
Contraceptives make women unhealthy, *N* = 250	49	19.6	68	27.2	48	19.2	51	20.4	34	13.6
Pregnancy must be properly planned, *N* = 250	133	53.2	28	11.2	11	4.4	73	29.2	5	2.0
Contraceptives interfere with sexual relationship between partners, *N* = 250	70	28.0	93	37.2	36	14.4	42	16.8	9	3.6
Contraceptive method results in infertility, *N* = 250	73	29.2	63	25.2	67	26.8	23	9.2	24	9.6
Women are more responsible in the use of modern contraceptives, *N* = 250	48	19.2	78	31.2	28	11.2	48	19.2	48	19.2
Contraceptive method prevents STIs, *N* = 250	151	60.4	22	8.8	11	4.4	52	20.8	14	5.6
Contraceptive method prevents pregnancy, *N* = 250	159	63.6	35	14.9	18	7.2	23	9.2	15	6.0
Contraceptive method helps in child spacing, *N* = 250	8	3.2	108	43.2	25	10.0	35	14.0	74	29.6

STI, sexually transmitted infection.

### Use of modern contraceptive methods, perceived barriers and side effects

About 38.3% had used modern contraceptive method before. A majority (76.7%) used male condoms as contraceptives. All 100% of those who have used contraceptives before used it to prevent pregnancies. About half of respondents (49.6%) mentioned weight gain as the main side effect of using modern contraceptives and 48.3% stated their religions encouraged the use of modern contraceptives. Slightly over a third (36.0%) stated that they were not using contraceptives because of the fear of stigmatisation (Table 4).

**TABLE 4:** Participants’ use of modern contraceptive methods, perceived barriers and side effects.

Variable	*n*	%
**Have you used modern contraceptive methods**		
Yes	133	38.3
No	214	61.8
**Which contraceptive methods have you used**		
Implant	6	4.5
Male condom	102	76.7
Pills	31	23.3
Injectables	52	39.1
Female condom	7	5.3
**Reason for using modern contraceptive**		
To avoid pregnancy	113	85.0
To prevent STIs	74	55.6
**Which contraceptive method are you currently using**		
Implant	12	4.6
Daily pills	16	6.1
injectables	21	8.0
Male condom	59	22.4
None	57	21.7
**What stops you from getting modern contraceptive methods**		
I don’t know anything about contraceptives	75	23.1
Long distance to facilities	26	8.0
Societal norms or prejudices	61	18.8
Stories you have heard about effects of contraceptive methods	47	14.5
Service provider’s attitude	45	19.1
I am afraid of stigma	117	36.0
I am afraid of side effects	49	15.1
I am afraid of infertility	31	9.5
It reduces sexual pleasure	28	8.6
They are not allowed in the school	7	2.2
I am afraid of my parents/partner	41	12.6
Nothing	29	8.9
Others	9	2.8
**What are the side effects of modern contraceptive methods**		
Heavy bleeding	97	28.0
Irregular bleeding	83	23.9
Absent menses	69	19.8
Abdominal cramps	22	6.3
Weight gain	173	49.6
Weight loss	57	17.5
Headaches	54	15.5
Dizziness	41	11.7
Nausea	14	4.0
Others	40	11.5
**Does your religion encourage the use of contraceptives**		
I don’t know	154	44.5
Yes	167	48.3
No	25	7.2

STI, sexually transmitted infection.

### Sexual activity of participants

Of the 250 participants, 144 (57.6%) reported being sexually active, while 106 (42.4%) were not.

#### Participants knowledge and current use of modern contraceptives

Among the 250 participants who had knowledge on modern contraceptives, 106 were using one or two methods of modern contraception while 144 participants were not on any form of modern contraception. Majority of participants using modern contraceptives are aged between 26 and 30 years while majority of those not using modern contraceptives were from the age group of 16–20 years. For education level, the majority of the participants who were currently using any form of modern contraceptives were junior secondary school students. A third of those were using modern contraceptives among the various types of disabilities were those with visual impairment. There was a statistically significant association between knowledge and current use except for the place of residence where the *p* is greater than 0.05 (Table 5).

**TABLE 5:** Participants’ knowledge and current use of modern contraceptives.

Variable	Current use				*p*-value	*χ* ^2^
	Yes (*n* = 106)		No (*n* = 144)			
	*n*	%	*n*	%
**Age group (years)**					< 0.001	80.3
10–15	0	0.0	3	2.1	-	-
16–20	10	9.4	92	63.9	-	-
21–25	38	35.8	18	12.5	-	-
26–30	58	54.7	31	21.5	-	-
**Marital status**					0.002	12.9
Married	16	15.1	7	4.8	-	-
Single	90	84.9	129	89.6	-	-
Widowed	0	0.0	8	5.5	-	-
**Education level**					< 0.001	49.7
Lower primary (1–4)	3	2.8	27	18.8	-	-
Upper primary (5–7)	27	25.5	19	13.2	-	-
Junior secondary	41	38.7	74	51.4	-	-
Senior secondary	19	17.9	16	11.1	-	-
Tertiary	0	0.0	8	5.6	-	-
Vocational training	16	15.1	0	0.0	-	-
**Occupation**					< 0.001	25.3
Unemployed	23	21.7	36	25.0	-	-
Artisan	1	0.9	0	0.0	-	-
Student	56	52.8	100	69.4	-	-
House wife or homemaker	8	7.5	0	0.0	-	-
Small business person	13	12.3	8	5.6	-	-
Junior civil servant	5	4.7	0	0.0	-	-
**Place of residence**					0.440	1.6
Rural	21	19.8	30	20.8	-	-
Urban	63	59.4	93	64.6	-	-
Settlement	22	20.7	21	14.6	-	-
**Region of residence**					< 0.001	38.6
South East or Southern	35	33.0	57	39.6	-	-
Kweneng	0	0.0	34	23.6	-	-
Kgatleng	33	31.1	25	17.4	-	-
Ngwaketsi or Kgalagadi	30	28.3	22	15.3	-	-
North East	4	3.8	1	0.7	-	-
North West or Ngamiland	4	3.8	5	3.5	-	-
**Religion**					0.001	13.4
African traditional religion	7	6.6	0	0.0	-	-
Christianity	92	86.8	141	97.9	-	-
Non-religious	7	6.6	3	2.1	-	-
**Type of disability**					< 0.001	56.4
Albinism	0	0.0	1	0.7	-	-
Deaf or hard of hearing	12	11.3	44	30.6	-	-
Physical disability	25	23.6	64	44.4	-	-
Speech impairment	23	21.7	0	0.0	-	-
Vision impairment	43	40.6	33	22.9	-	-
Multiple impairments	3	2.8	2	1.4	-	-

## Discussion

The main aim of this study was to assess the knowledge, attitude and factors associated with uptake of modern contraceptive methods among young women living with disabilities in Botswana.

Most of the participants who reported knowledge of contraception were in the age group 26–30 years, had more years of formal education, live in urban areas and has physical or visual impairments. Some of the unfavourable attitudes expressed were that contraceptives make women unhealthy, interfere with sexual relationship and result in infertility. Two fifths reported current use with the male condom being the most commonly reported. Reported barriers to use of contraception were fear of stigma, service provider’s attitude and societal norms while the most common reported side effects were menstrual abnormalities and weight gain. Finally, most participants not using modern contraceptives were single, Christians, with hearing or physical disabilities and resided in the South east and Kweneng districts.

Most of the study participants were in 16–20 years and 26–30 years age groups, highlighting a pivotal stage in reproductive health awareness and decision-making. The findings from this study is different from a work from Tanzania^11^ and Uganda^12^ among university students where the majority of the participants were aged 20–24 years. The results from this study show older participants (26–30 years) exhibited a higher awareness level of modern contraceptive methods compared to their younger counterparts. This suggest the potential impact of age-related experiences, access to information over time and evolving societal attitudes towards reproductive health. About 91.1% of the participants were single. This was similar to the findings in Uganda^12^ among undergraduate students, where 87.5% of the participants were single. The predominance of single participants (91.1%) underscores the autonomy these women hold in their reproductive choices. The findings from this study indicate marital status as a significant factor influencing attitudes towards contraception.

The majority of the respondents (69.50%) were students with one-third (35.2%) in junior high school while 24.0% were unemployed. Educational background plays a pivotal role in contraceptive prevalence.^13^ This finding is in consonance with some studies from a work in Ghana^14^ and Uganda.^15^ Individuals living with disabilities who received primary, secondary and tertiary education are more likely to engage in contraceptive use than those without any formal education. This could be attributed to education serving as the crucial factor in seeking information and dispelling traditional beliefs and unfounded rumours regarding the side effects and safety of contraceptive methods.^16^

Over half of the participants lived in the urban regions while majority were Christians. This aligns with the studies elsewhere,^11,12,17,18^ where the majority of participants were Christians. Regional differences emphasise the importance of recognising regional nuances in crafting targeted interventions. Many have also noted the potential influence of religious beliefs on attitudes towards contraception and its uptake.^18^ A study in Uganda identified religious beliefs as a key factor limiting the use of modern contraceptive methods.^12^ This finding underscores the need for sensitive, context-specific approaches that respect diverse religious perspectives.

The commonest disabilities among the participants were impairment of hearing (36.7%) and physical disabilities (30.1%). According to Horner-Johnson et al. (2019), women living with disabilities frequently lack knowledge about contraceptives and may opt for a more restricted range of contraceptive methods. In particular, women who are deaf or hard-of-hearing exhibit lower knowledge about contraceptive methods.^19^

According to Beyene et al., participants with hearing impairments and visually impaired participants were six times more likely to use modern contraceptives. One explanation could be that, in contrast to hearing-impaired women, women with visual impairments had more access to information about contraceptives on radio channels. It has been observed that radios are the most efficient means of reaching vulnerable and marginalised populations with information about changing their behaviour and of removing cultural barriers to the use of modern contraceptives.^20^

This study revealed that a substantial majority had heard of contraceptive methods, with health personnel being the primary source of information for nearly 70% of the participants. This finding is similar to the 78.3% of participants who reported hearing about modern contraceptive methods from health facilities in a study conducted among university students in Botswana^21^ but differs from the study conducted in Ethiopia^13^ where it was noted that the most common source of information was the media. The percentages of participants that have source of information from radio and television indicate that despite the disabilities, they are exposed to the media, a finding consistent with a study in Ethiopia.^10^ This shows that the media can play an important role in the uptake of contraception among people living with disabilities, and hence there should be educational programmes in the media on safe use of contraceptives.

The oral pills (60.4%) were the contraceptive method that was recognised by the largest proportion of participants, consistent with a work in southwest Nigeria.^17^ A significant percentage of the participants (59.2%) knew the injectable method similar to the findings in Ethiopia,^22^ where the dominant modern contraceptive method known was the injectable method. The male condom was recognised by 56.6%. This may be on account of its role of preventing STIs. A majority of the participants indicated they used modern contraceptives to prevent STIs (81.6%). Other reasons for using modern contraceptives are for stopping child birth (52.0%), limiting number of children (41.6%) and spacing of birth interval (27.6%).

Our findings revealed disturbing gaps and a need for urgent interventions to address myths and misconceptions about reproductive health issues, including contraceptives, among these young people living with disabilities in Botswana. More than a third believed that contraceptives make women unhealthy, interfere with sexual relationships and result in infertility and that women are more responsible for modern contraceptives. In addition, more than two in five did not know or disagreed that contraceptives prevent pregnancy or helped in child spacing. Participants from this study (54.4%) indicated that contraceptives result in infertility, whereas other study found out that only 18% agreed that contraceptives lead to infertility.^23^ To address these misconceptions, factual information on contraceptive use must be made available through campaigns, intense mobilisation on modern contraceptive methods.

Information and educational materials on contraception and reproductive health should be disability friendly (sign language, braille and in print and audio).

The most common modern contraceptive methods currently in use are the male condom (22.4%) and injectables (8.0%), with the implant being the least used. This is different from the findings of a similar study,^16^ where the most commonly reported methods in use among females living with disabilities were implants, followed by injectable (36%) and oral contraceptive pills (12%). Condoms (4%) were the least used method.^16^ The popularity of the male condom may be attributed to its accessibility, dual protection benefits (against both sexually transmitted infections and pregnancy) and ease of use. In contrast, the lower utilisation of the implant (4.6%) may be attributed to awareness, accessibility or personal preferences regarding long-acting hormonal methods.^24^

The most commonly reported reasons for not using modern contraceptives include lack of knowledge, societal norms and fear of stigmatisation. These findings are consistent with the study conducted in sub-Saharan Africa.^5^ The reasons for the underutilisation of contraceptives among people living with disabilities include a lack of education and knowledge regarding sexual and reproductive health services, inadequate treatment by healthcare workers and the presence of healthcare facilities and services that are not accessible to individuals living with disabilities.^5^ Studies in India have reported similar findings.^25^ It is clear that person with disabilities (PWDs) face barriers to accessing contraceptive services in Botswana. To achieve the Sustainable Development Goal 3 objectives in Botswana,^26^ which focus on universal and/or fair access to thorough sexual, reproductive and maternal health services, immediate and context-specific policy measures and interventions tailored to address the identified barriers in this review are essential. ‘It is imperative to incorporate women and girls living with disabilities in the formulation of family planning policies and programmes, adhering to the principle of “nothing about us, without us”’. This involves actively collaborating with local disability service organisations to enhance accessibility.^27^ Educational messages and awareness-raising on modern contraceptives should be tailored to the needs of young person living with disabilities. In addition, there is a need to address negative attitudes among healthcare workers through training and making sure that modern contraceptives are available for women and girls living with disabilities.^27^

The majority of the participants from this study identified weight gain (49.6%) as the most leading side effects of using modern contraceptives followed by heavy and irregular bleeding. This is comparable to the findings in a study in sub-Saharan Africa,^28^ which reported that young women may face personal barriers to contraceptive use because of known side effects, such as weight gain, bleeding, high blood pressure, headaches and disruptions in the menstrual cycle. It has been reported that women reported experiencing a range of adverse effects including serious ones like blood clots and that the side effects are linked to the use of modern contraceptives.^29^ Mood fluctuations, recurrent yeast infections and breast soreness were among the less severe side effects. Women in all groups believed that they had not received enough information regarding possible adverse effects of birth control when receiving them at the healthcare facilities.^30^

These young people would continue to avoid using contraceptives if these anxieties are not effectively managed and women are not reassured of the crucial benefits of doing so, which would not be good for the attainment of universal health coverage.^31^

### Strengths and limitations

A key strength of the study is its focus on a marginalised population, young women living with disabilities providing valuable insights into their unique barriers to accessing modern contraceptive methods with representation of most disability types, an area often overlooked in reproductive health.

A significant limitation of the study was that some of the organisations providing services to people living with disabilities did not grant permission to conduct the research with their participants. This restricted the diversity of organisations included in the study.

### Recommendations

#### Recommendations for policy

**Tailored reproductive health education programmes:** Recognising the diversity within the demographic landscape, there is a pressing need for tailored reproductive health education programmes. These programmes should be designed to cater to the specific needs of young women living with disabilities, considering factors such as age, educational background and type of disability.

**Accessible contraceptive services:** Policy initiatives should focus on improving the accessibility of contraceptive services, particularly in rural and settlement areas. This involves locating healthcare facilities, ensuring the availability of different modern contraceptive methods and addressing challenges faced by women living with disabilities. Health authorities should work hand in hand with disability advocacy groups to facilitate the development of inclusive service delivery models.

**Inclusive family planning policies:** Policy frameworks should explicitly incorporate the unique needs of young women living with disabilities within family planning initiatives. This includes financial support for contraceptives, addressing the barriers and involving the disability representatives in the development and evaluation of family planning policies.

**Support for comprehensive sexuality education:** Integrating comprehensive sexuality education into school curricula is paramount. This not only addresses the knowledge gaps identified in the study but also contributes to fostering a culture of open communication and dispelling myths surrounding contraception. Policymakers should advocate for the inclusion of comprehensive sexuality education that is accessible and adaptable to the diverse needs of students living with disabilities.

#### Recommendations for health service delivery or practices

**Inclusive training for healthcare providers:** Healthcare providers should undergo specialised training to enhance their understanding of the unique needs and challenges faced by young women living with disabilities. This training should encompass effective communication strategies, cultural sensitivity and the provision of inclusive care.

**Utilisation of assistive technology:** Incorporating assistive technologies within healthcare settings can significantly enhance accessibility. From text-to-speech applications to sign language interpretation services, leveraging technology can bridge communication gaps and ensure that individuals living with disabilities receive information in a format that suits their needs. Facilities should invest in and promote the use of these technologies.

#### Recommendations for further research

**Longitudinal studies on reproductive health dynamics:** They can provide a deeper understanding of how knowledge, attitudes and contraceptive practices evolve over time among young women living with disabilities. Tracking changes in these dynamics can offer valuable insights into the long-term impact of interventions, societal shifts and individual experiences, contributing to a more nuanced understanding of reproductive health issues.

**Evaluation of intervention programmes’ efficacy:** Assessing the efficacy of the existing intervention programmes and policies is very important. Researchers should conduct comprehensive evaluations of initiatives aimed at improving reproductive health knowledge, accessibility of services and attitudes towards contraception among young women with disabilities. This evaluation can provide evidence-based insights into the impact of interventions and guide future programme development.

#### Technological innovations for accessible healthcare

Research focusing on the integration of technological innovations to enhance healthcare accessibility is warranted. Exploring the efficacy of mobile applications and other digital platforms in delivering reproductive health information and services to young women and girls living with disabilities can be instrumental in overcoming barriers.

## Conclusion

The aim of this study was to assess the knowledge, attitudes and factors associated with the uptake of modern contraceptive methods among young women living with disabilities in Botswana. The socio-demographic profile of the 349 participants revealed a diverse landscape shaped by factors such as age, marital status, education and region of residence. The level of knowledge varied among participants with majority having heard of contraceptive methods, primarily from health personnel. Our findings revealed disturbing gaps and a need for urgent interventions to address myths and misconceptions about modern contraceptives among these young people living with disabilities in Botswana. The recommendations for policy and health service delivery practices were provided with focus on inclusivity, education and dignity.

The research marks a significant waypoint, inviting a continual exploration, advocacy and transformative change. The conclusion is not a final chapter to action. The future scholars, policymakers and practitioners are to build upon this foundation. The research journey concludes but the mission for reproductive health equity endures.
